# Reliability analysis of the solidification cooling of solid rocket motor grain material

**DOI:** 10.1371/journal.pone.0306208

**Published:** 2024-08-20

**Authors:** Juan Du, Yangtian Li, Yun He

**Affiliations:** 1 College of Statistics and Mathematics, Inner Mongolia University of Finance and Economics, Hohhot, Inner Mongolia, China; 2 College of Water Conservancy and Civil Engineering, Inner Mongolia Agricultural University, Hohhot, Inner Mongolia, China; UNICAMP, University of Campinas, BRAZIL

## Abstract

The reliability of solid rocket motor grain structure during solidification cooling is analyzed. First, a three-dimensional parametric modeling of the grain is carried out by ANSYS finite element software. The dangerous point and dangerous moment can be obtained based on the transient and dynamic thermo-structure coupling under the cooling condition. Moreover, the maximum equivalent strain and temperature values are extracted. Second, a dual neural network model is established based on the probability distribution of the copula function and specific parameters. Finally, the instantaneous reliability during the solidification cooling process of the grain is calculated. Then, the dynamic reliability analysis is realized. The proposed method reduces the computational cost of dynamic reliability of grain structure, demonstrating its applicability in practical engineering problems. Furthermore, comparing the results of the proposed method with the MCS method demonstrates that the proposed method has high computational accuracy.

## Introduction

The reliability of a structure is closely related to people’s lives and property safety. Therefore, scholars have conducted in-depth research on structural reliability. Shadab et al. proposed a step-by-step algorithm based on the advanced first-order second moment method to calculate the failure probability and sensitivity analysis of structures. They applied the method to geotechnical engineering and verified its effectiveness [1]. The ANFIS model is obtained by combining neural networks and fuzzy logic. Ambassa et al. used ANFIS to model the evolution process of dam water level. Then, they evaluated the stress state on the structure and the slope stability under shear based on hydraulic behavior. The results indicate that the model provides satisfactory results [2]. Dai et al. proposed a method of digital image processing and applied it to the reliability evaluation of highway tunnel services. They demonstrated that this evaluation method has the advantages of strong visibility, simple evaluation methods, and being beneficial to engineering practice [3]. Huang et al. embedded the reliability of pipeline network nodes into the reliability of edges based on general connectivity probability analysis algorithms. They calculated the reliability of each node in different soil layers using the finite element numerical simulation method based on the reliability calculation theory of various pipeline components. This study lays the foundation for further accurate calculation of pipeline network connectivity [4].

The grain material is an important part of the solid rocket motor. Moreover, grain is a viscoelastic material. Its mechanism of stress deformation is very complex. In recent years, the reliability analysis of grain material has been widely discussed by scholars.

Yilmaz et al. established the mathematical model of the grain material’s stress and strain by using the response surface method. Then, they constructed the limit state function. They also used the first-order reliability method to analyze the instantaneous reliability within the confidence interval [5]. Shen et al. proposed a numerical calculation model combining the Voronoi cell finite element method with the homogenization method. This model can evaluate the mechanical performance parameters of solid rocket motor grain [6]. Liu et al. proposed using the fracture elongation of propellant as the judgment basis for the failure of grain material under working internal pressure. Moreover, they used the finite element analysis of the grain material to prove the accuracy of the criterion further [7]. During the processes of curing and cooling, ignition, and pressure rise, two situations occur between the grain and the shell: binding and nonbinding. These situations lead to different stress conditions of the engine. Considering this situation, Deng et al. studied the structural integrity of the grain based on maximum strain energy theory [8]. Zhou et al. used the stress–strength interference model to calculate the variation trend of the reliability of the grain material with the strain sensitivity coefficient. Based on this, the influence of constant strain on the probability storage life of engine grain is analyzed [9]. Wang et al. conducted the reliability study of solid rocket motor grain based on the life cycle load to study the effect of vertical storage on the storage reliability of solid rocket motor grain. Their study showed that the relationship between the ignition reliability and the number of vertical storage can be expressed by a negative exponential function [10]. Li et al. performed the structural integrity simulation and experimental research of the motor charge to evaluate the safety factor of a solid motor charge under low-temperature ignition conditions. They obtained the maximum elongation of the propellant under the simulated low-temperature ignition condition by combining the low-temperature fast and slow combination tensile test of the grain. Then, the safety factor of the engine grain under a low-temperature ignition condition is obtained [11].

In the above grain material analysis, considering the correlation of grain material parameters complicates the problem. Therefore, the researchers did not consider the correlation between the material parameters of the grain. Grain is an important part of the solid rocket motor. The grain material’s reliability is directly related to the overall reliability of the rocket engine. Considering the correlation between performance parameters is the key to the accurate analysis of the grain material’s reliability.

Cracks may occur on the inner surface of the grain during the solidification cooling process of the grain material. At the light level, cracks can change the thrust characteristics of the engine; at the heavy level, they can cause explosion and other accidents. Therefore, this study investigates the reliability of the calculation for the solid rocket motor grain material’s solidification cooling.

In the study, an ANSYS finite element analysis model is constructed based on the geometric and performance parameters, boundary conditions, and temperature loads of the grain material. When the charge is solidified and cooled, the instantaneous reliability of the grain’s dangerous point at the dangerous moment is calculated. It is used to measure the grain’s reliability during the whole curing process. The joint probability density function between variables is constructed through the copula function. The dual neural network direct integration method is applied to calculate the instantaneous reliability of the dangerous point at the dangerous moment during the solidification cooling process of the grain. Then, the reliability index of the inner surface crack of the grain is solved.

## Description of grain material solidification cooling

**Fig 1:**
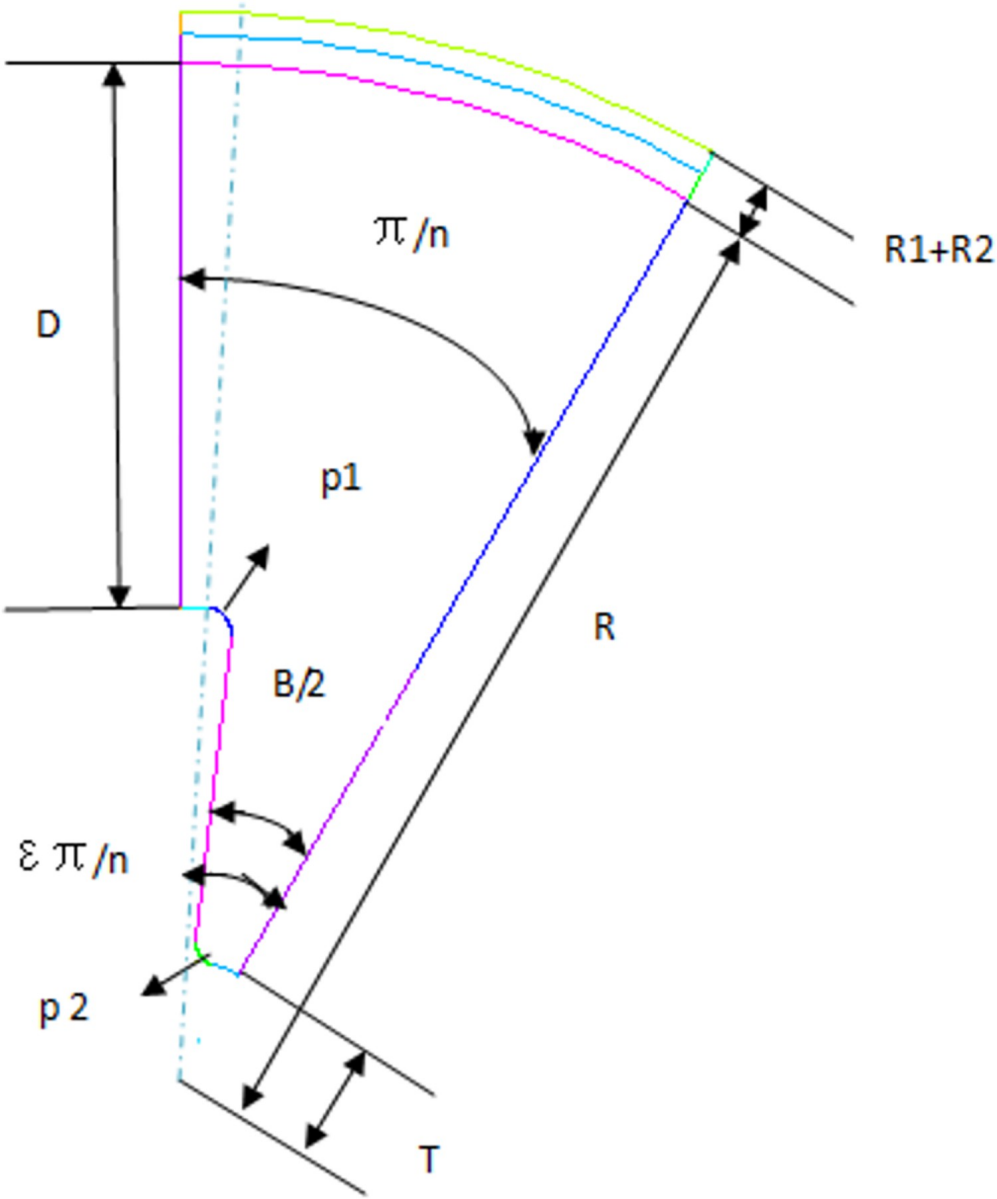
Cross section of grain.

The middle section of a solid rocket motor is composed of a shell, an insulating layer, and a hexagonal star grain. The grain material is an IPDI-HTPB propellant. Only 1/12 of the grain structure’s periodic symmetry model is established because the engine geometry and load are symmetrical, as shown in Fig 1.

The structural geometric parameters are as follows: column length (*L*) of 1600e-3 m; shell thickness (*R1*) of 1.5e-3 m; thickness of thermal insulation layer (*R2*) of 2.7e-3 m; outer radius of the charge column (*R*) of 159.1e-3 m; internal radius (*T*) of 22.5e-3 m; wall thickness (*D*) of 105e-3 m; star angle number (*n*) of 6; circular arc radius of star groove (*P1*) of 5e-3 m; the included angle of the star side (*B*) of 47°; star angle coefficient (*ε*) of 0.8; radius of the star heel arc (*P2*) of 4e-3 m.

The reliability of the crack failure on the inner surface of the grain is analyzed during the process of cooling from the curing temperature of 65°C to the ambient temperature of 20°C.

## Finite element modeling of the grain structure

**Fig 2:**
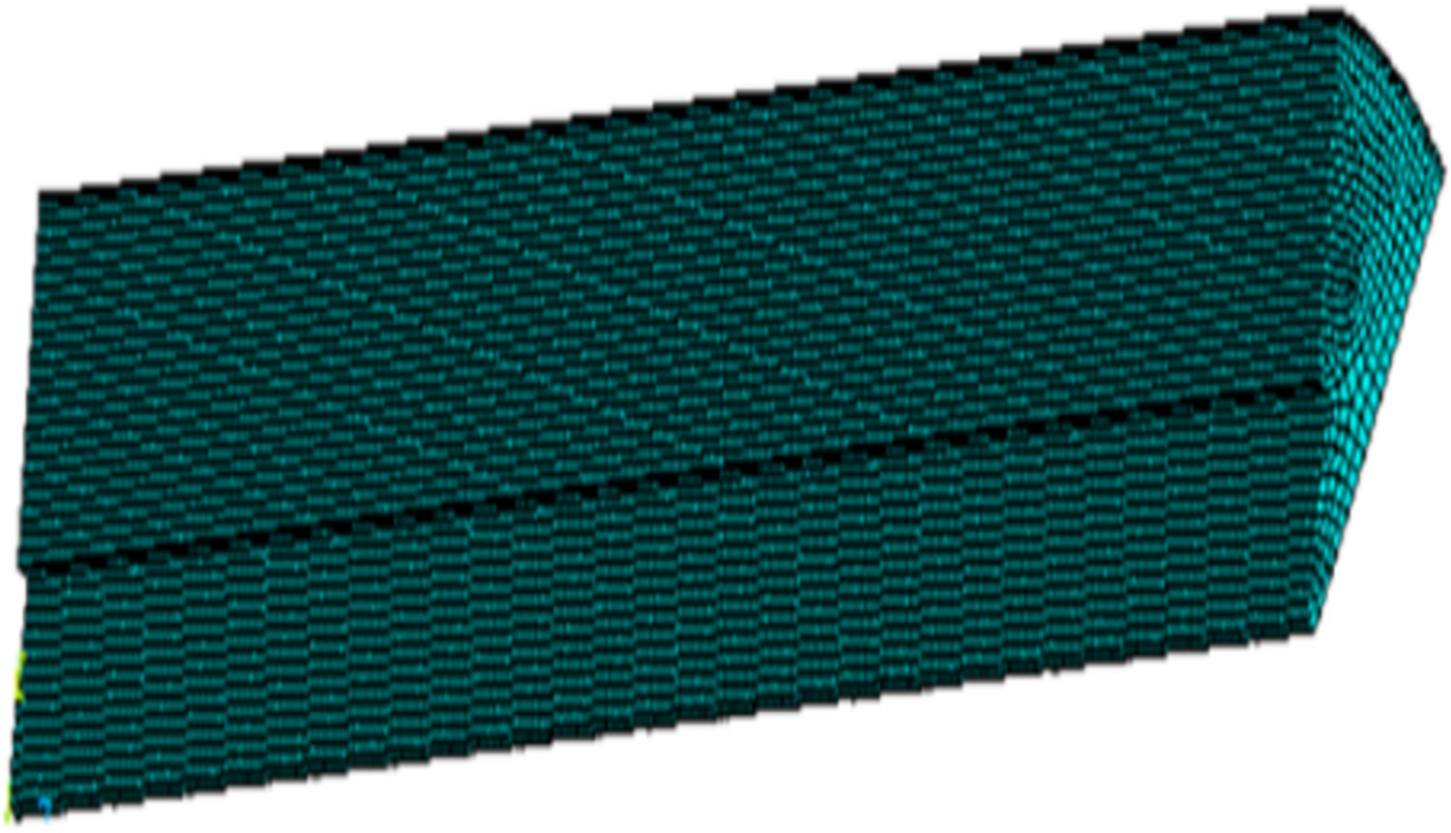
3D finite element model of grain.

Based on the geometric model of the grain structure, the finite element model of the structure is built using ANSYS software. The hexahedral coupling element SOLID5 is used for the construction, as shown in Fig 2. The specific material parameters are shown in Table 1.

**Table 1:** Material performance parameter of the grain, insulation layer, and case.

Parameters	Grain	Insulating layer	Shell
Modulus / MPa	*E*(t)	24.178	1.9610e5
Poisson’s ratio	0.49602	0.49602	0.29001
Density / kg·(m^3^)^−1^	1.7500e3	1.2800e3	7.8500e3
Specific heat capacity / J·(kg·k) ^−1^	1256.1	2261.1	512.91
Linear expansion coefficient / k^−1^	2.3301e-4	2.3346e-4	1.1000e-5
Thermal conductivity / w·(m·k) ^−1^	0.45000	0.29001	27.633

When the reference temperature is 20.12°C (293.27 K), the WLF equation of the time–temperature displacement factor can be written as

$$\mathrm{lg}a_{T}(T)=\frac{-5.27(T-293.27)}{145.2+T-293.27}.$$ (1)

After the fitting by Prony series, the relaxation modulus of the grain can be written as

$$E(t)=E_{\infty }+\sum_{i=1}^{N}E_{i}\cdot \exp(-t/\tau_{i}),$$ (2)

where *E* is the equilibrium modulus. The coefficients of E (t) are shown in Table 2.

**Table 2:** Prony series of the IPDI-HTPB propellant.

*i*	1	2	3	4	5	6
*E*_*i*_/MPa	8.8331	4.1438	0.53667	0.37647	0.08331	0.35124
*τ*_*i*_/s	0.0038000	0.11020	3.5894	99.931	3070.1	94351

The outer surface of the grain is assumed to bond completely with the inner surface of the thermal insulation layer. Moreover, the outer surface of the thermal insulation layer presumably bonds completely with the inner surface of the shell. The inner hole surface is free. Applying the corresponding symmetry constraints the symmetry plane, and the normal displacement is zero. The shell is not deformed because the elastic modulus of the shell is far greater than the relaxation modulus of the grain. Full restraint is applied to the shell’s outer surface. During cooling, the engine dissipates only the heat from the outer wall, and the rest is insulated.

For the temperature load, the grain is solidified after pouring. Its zero stress temperature is 65°C. After 100 h, the grain is slowly cooled linearly to 20°C and kept constant at 20°C. Given that the curing and cooling process of the engine is relatively slow, the temperature of the engine is assumed to decrease uniformly. In particular, the temperature in the whole engine at any instant is a uniform field.

## Calculating the reliability of grain solidification cooling

### Constructing copula function and training sample

During the solidification cooling process of the rocket engine, the Von Misses strain of the propellant increases rapidly with the increase in the linear expansion coefficient of the performance parameter. Moreover, the allowable elongation of propellant decreases with the decrease in temperature. Thermal conductivity is the main factor affecting the temperature change of propellant [12,13]. During solidification cooling, the linear expansion coefficient and the thermal conductivity are usually selected as the main influencing factors. The distribution characteristics of the two parameters are shown in Table 3.

**Table 3:** Distribution types of the thermal expansion and thermal conductivity coefficients.

Material parameters	Distribution type	Mean value	Standard deviation	Correlation coefficient
Linear expansion coefficient (k^−1^)	Normal distribution	2.33e-4	5.06e-5	0.83
Thermal conductivity (w/m·k)	Normal distribution	4.50e-1	1.35e-1

(1) Analyzing the dangerous points and dangerous moments of the grain material

**Fig 3:**
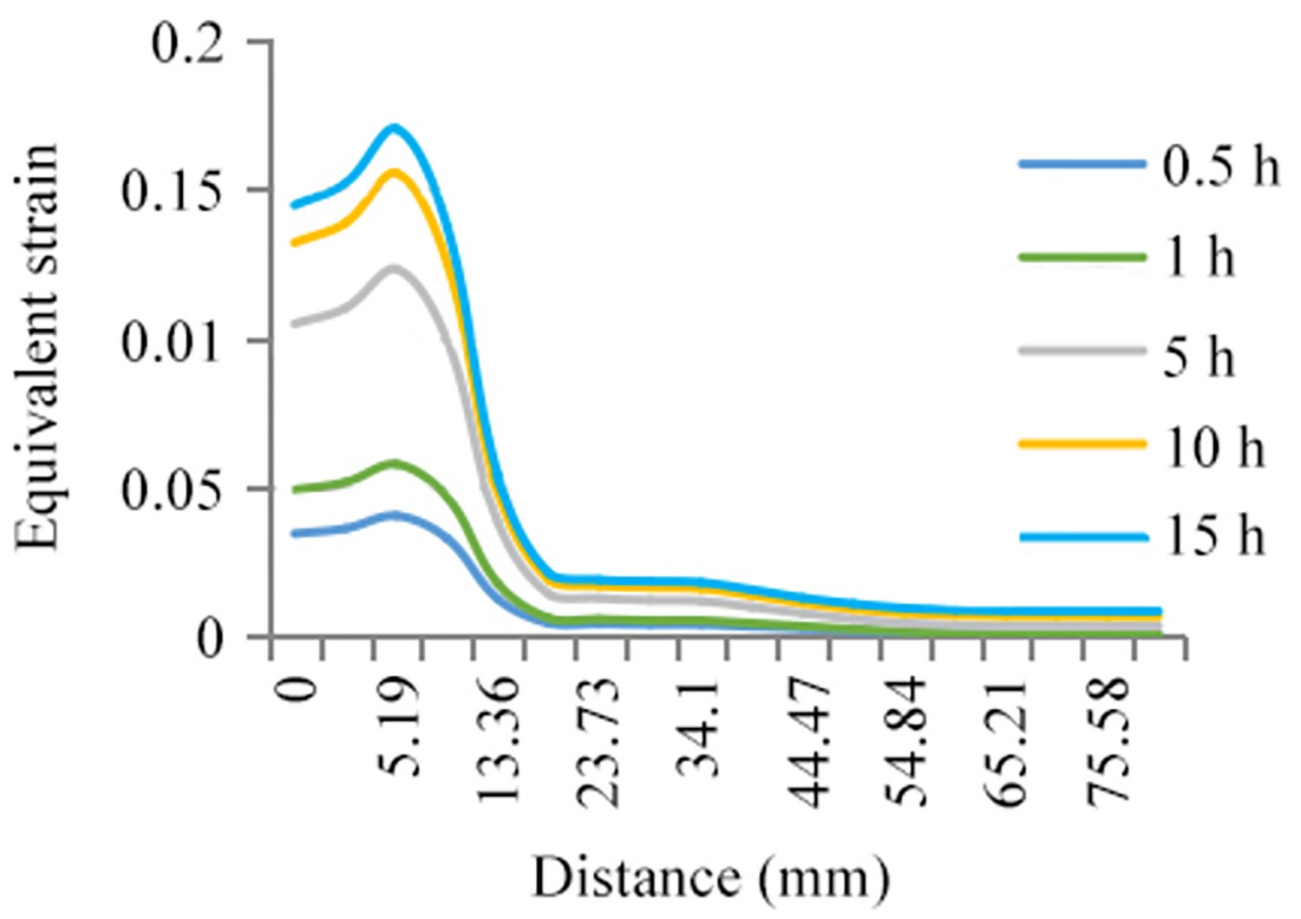
Equivalent strain curves of each node in a twelfth of grain inner surface at five different moments.

The linear expansion coefficient and the thermal conductivity coefficient are regarded as the effective input variables for the reliability analysis of grain solidification cooling. The mathematical expectation of the two variables is taken as the input variable and substituted into the finite element analysis software package. Then, the transient finite element analysis of the grain at different times is conducted. The equivalent strain curves of each node on the inner surface of the grain at different times are obtained, as shown in Fig 3. The figure shows that the strain response of the node located at 5.19 mm of the top of the inner surface always maintains the maximum at different times. This point is dangerous.

**Fig 4:**
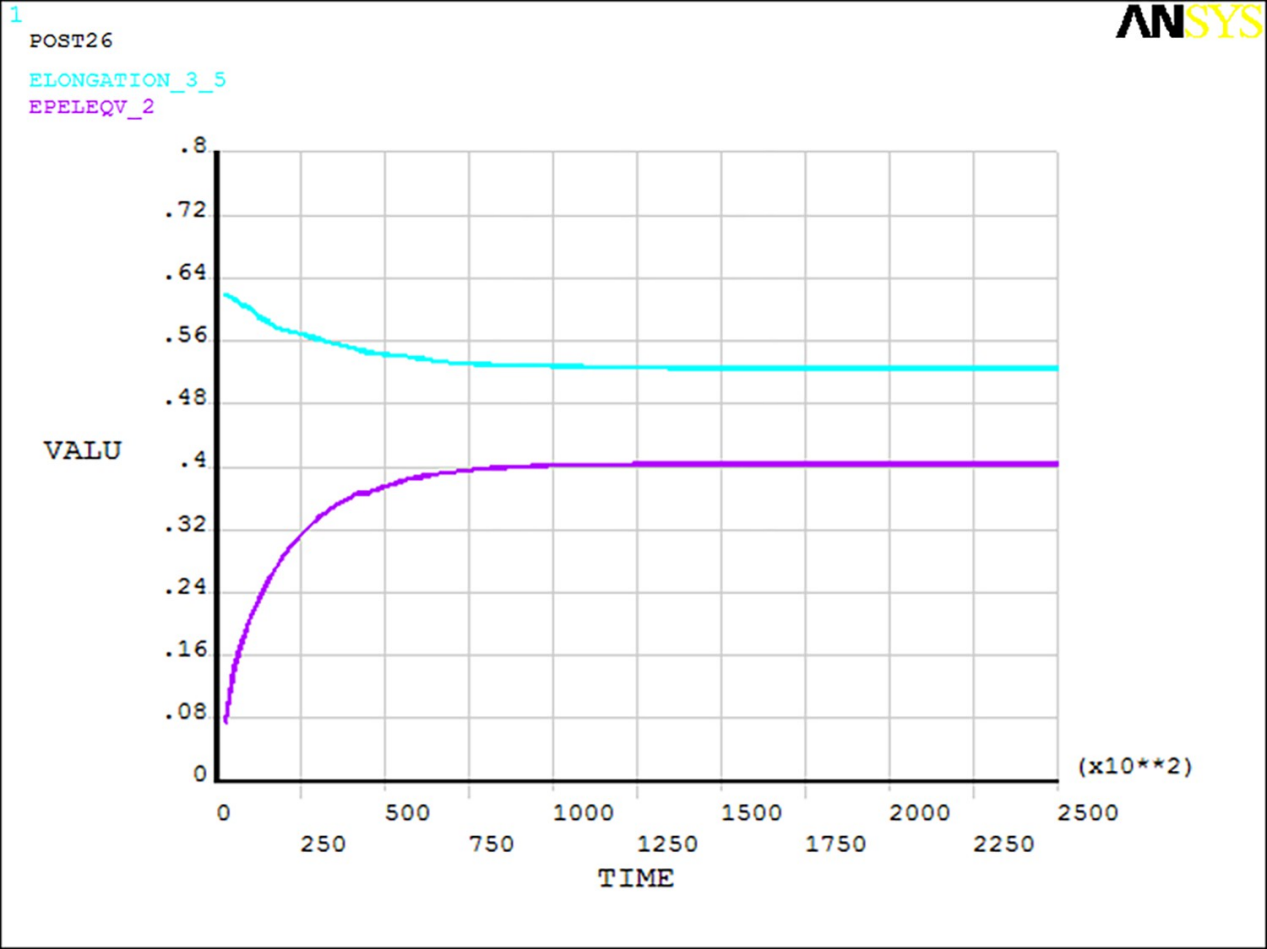
Curves of equivalent strain and elongation over time in the dangerous position.

The mathematical expectation of the two parameters is used as the input variable for dynamic finite element analysis. The equivalent strain and allowable elongation curve of the dangerous point with time can be obtained, as shown in Fig 4. The figure shows that the equivalent strain and allowable elongation at the dangerous point tend to be stable at 12e4(s). The difference between the equivalent strain and allowable elongation tends to be the smallest. Therefore, 12e4(s) is selected as the dangerous moment of reliability analysis.

(2) Constructing the copula function and generating training samples

**Fig 5:**
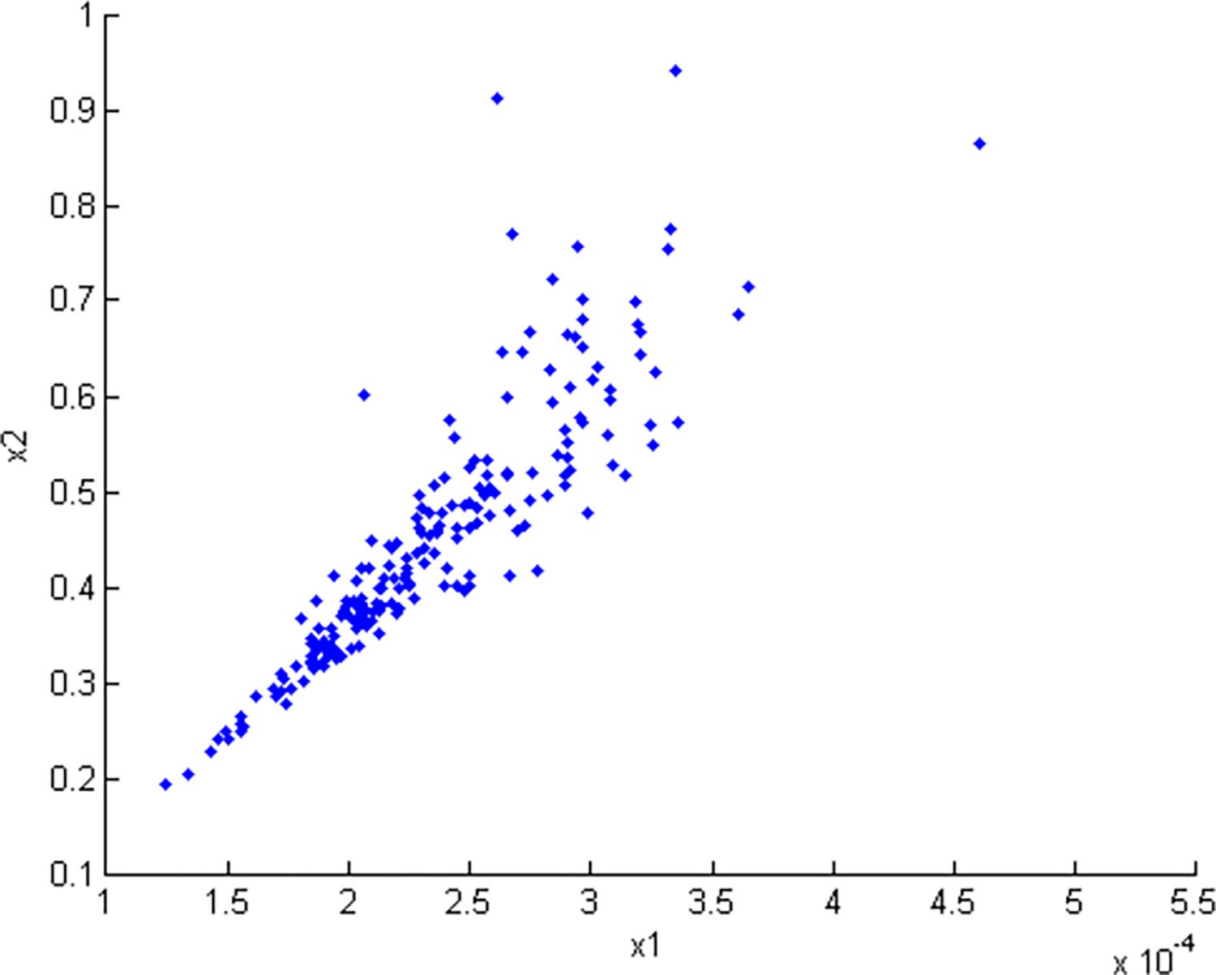
Scatter plot of the relevant variables *x*_1_ and *x*_2_ of Clayton Copula simulation.

**Fig 6:**
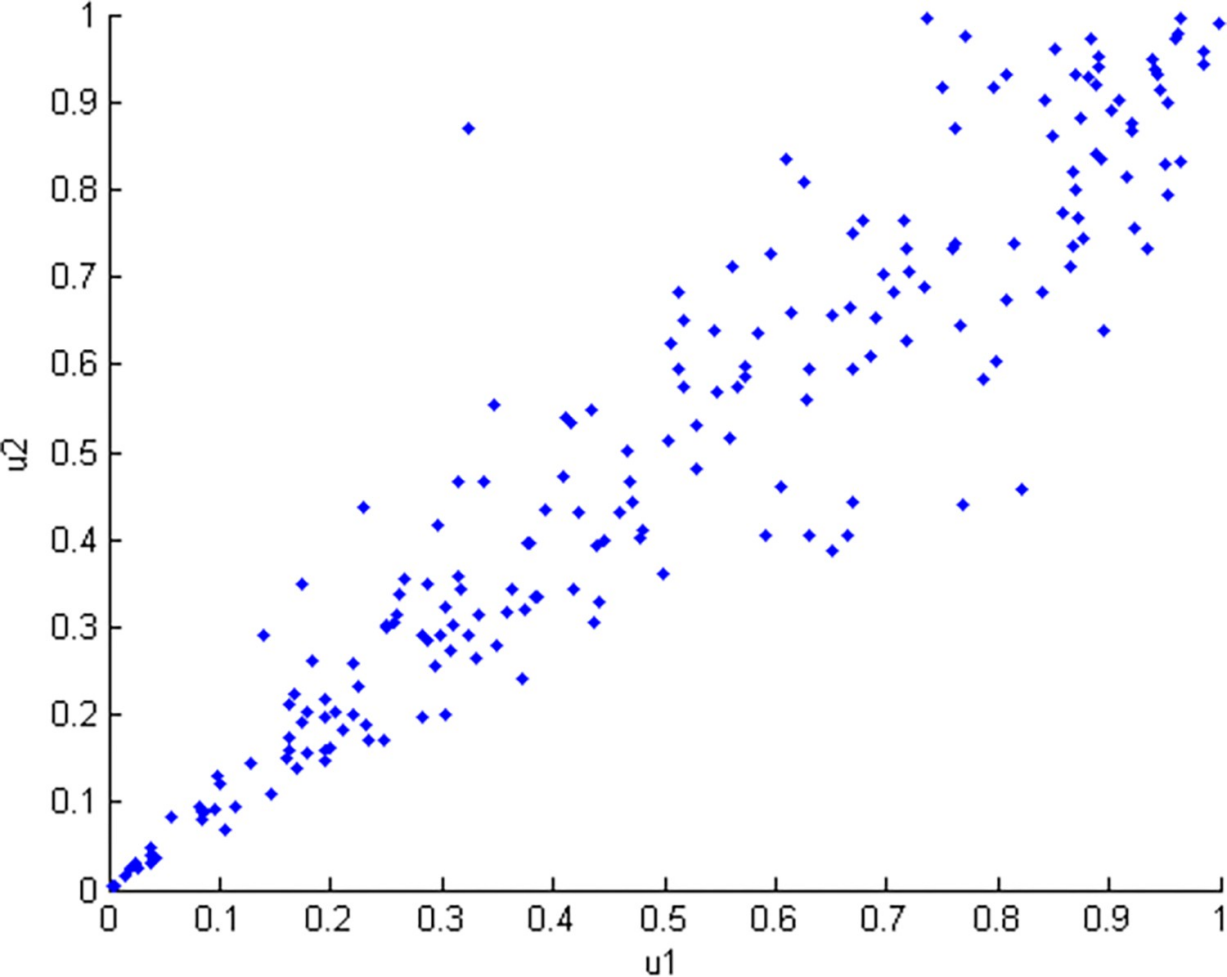
Scatter plot of u_1_ and u_2_ in the Clayton Copula function.

Sklar proposed copula theory, which states that any multidimensional joint distribution function can be decomposed into a corresponding edge distribution function and a copula function. This theory is widely applied [14,15]. In this study, the distribution characteristics of the linear expansion coefficient and the thermal conductivity coefficient indicate that their joint probability density function is constructed by the Clayton copula function. The correlation parameter *θ* = 7.15 between the two variables is obtained, thereby generating 200 sample points (*x*_1_,*x*_2_), as shown in Fig 5. Moreover, the scatter diagram of *u*_1_ and *u*_2_ in the Clayton copula function is given, as shown in Fig 6.

(3) Establishing the function of deformation and the failure of grain material

**Fig 7:**
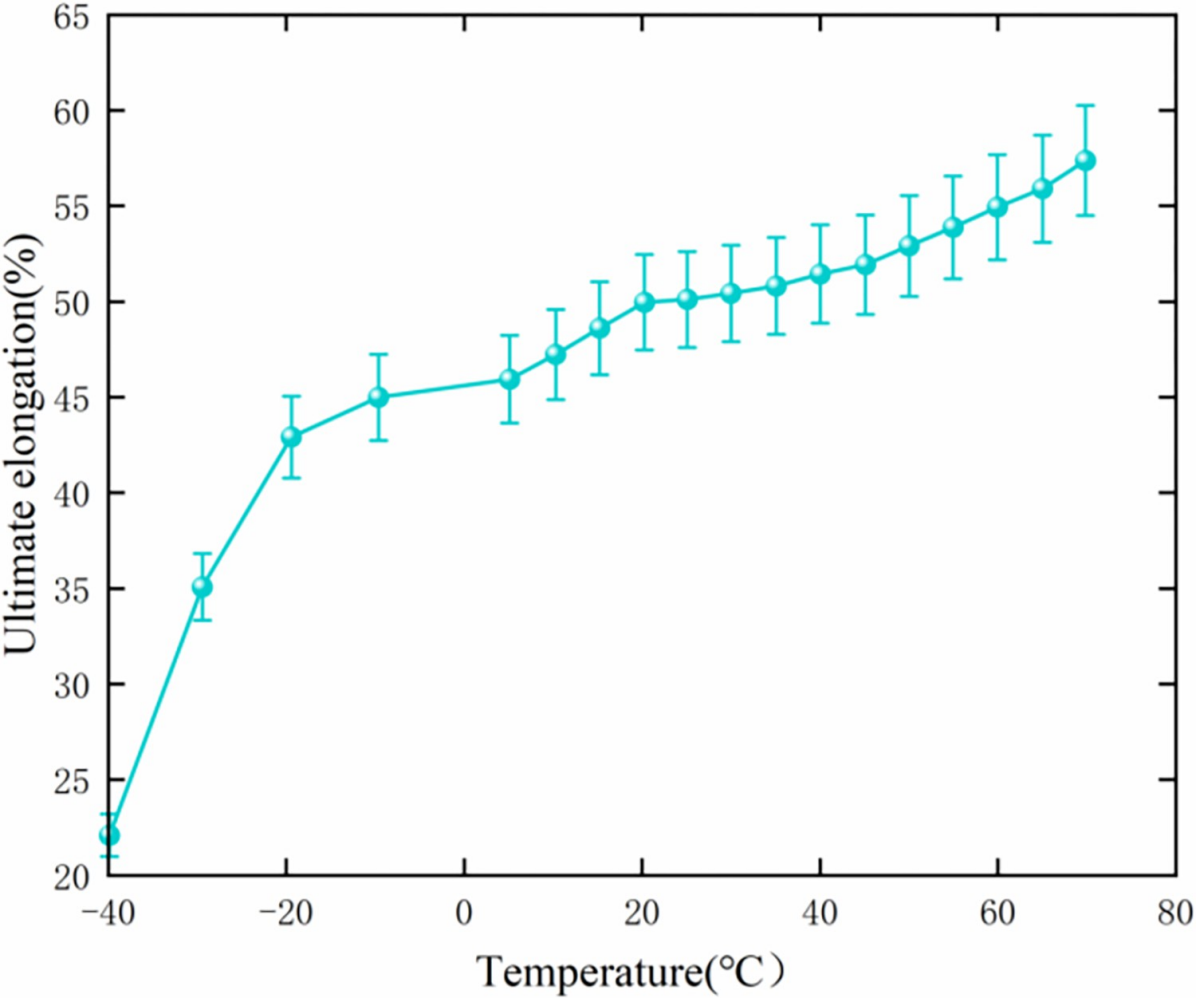
Ultimate elongation–temperature curve of IPDI–HTPB grain at the stretching rate of 20mm/min.

The variation curve of the allowable elongation with temperature [13] is shown in Fig 7. The samples of linear expansion coefficient and thermal conductivity in Fig 5 are substituted into the finite element analysis software package. The transient finite element analysis is conducted by considering the temperature value of the dangerous point at the dangerous moment of t = 12e4 (s). The allowable elongation sample *ε*_*s*_ and the maximum equivalent strain sample *ε* at the dangerous point can be obtained. Then, the function of deformation and the failure of grain material is established. In particular,

$$Z=\varepsilon_{s}-\varepsilon .$$ (3)

(4) Establishing the reliability expression of the grain inner surface

The calculation expression of the reliability of the grain inner surface can be expressed as follows through the above analysis:

$$\begin{array}{l} P=\int_{-\infty }^{+\infty }\int_{-\infty }^{+\infty }f_{X}(x_{1},x_{2})dx_{1}dx_{2}\\ \;Z>0 \end{array}$$

$$\begin{array}{l} =\int_{-\infty }^{+\infty }\int_{-\infty }^{+\infty }f_{1}(x_{1})f_{2}(x_{2})c(u_{1},u_{2};\theta )dx_{1}dx_{2}.\\ \;Z>0 \end{array}$$ (4)

### Calculation of structural reliability through the direct integration method based on dual neural network

In Eq (4), the reliability expression of the grain inner surface is a double definite integral. The integrand function is

$$y(x_{1},x_{2})=f_{1}(x_{1})f_{2}(x_{2})c(u_{1},u_{2};\theta )$$ (5)

The reliability of the grain inner surface is calculated by the direct integration method of the dual neural network [16–18]. The specific steps are as follows:

(1) Constructing the original function neural network

In the original function network, the vector form of the function relationship between output and input variables is

$$Y=w_{b}^{T}f(KX+b)+c.$$ (6)

**Fig 8:**
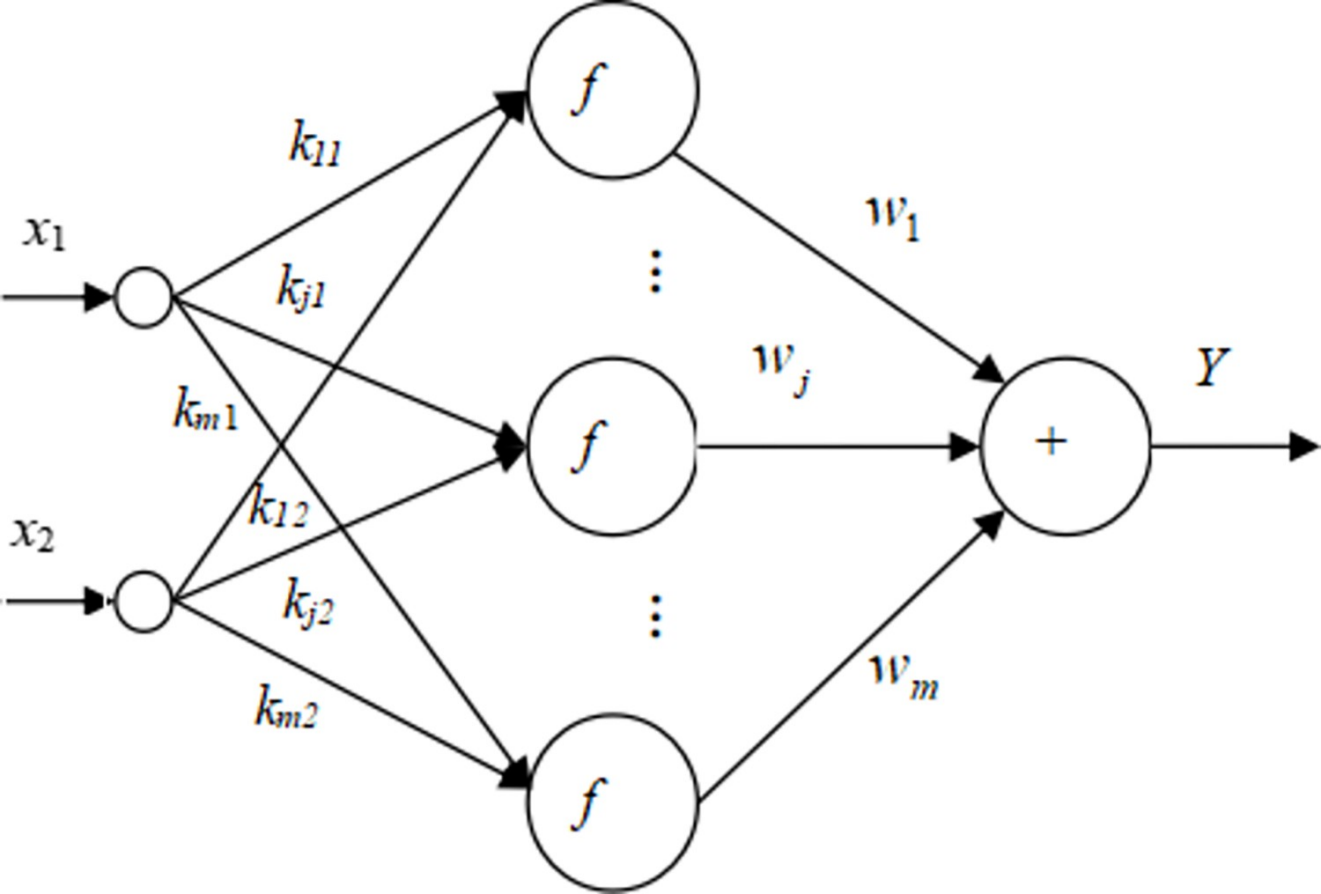
The original function network structure.

The original function network is a three-layer neural network comprising the input layer, the hidden layer, and the output layer where *f* = *e*^*x*^ is the activation function of hidden layer unit; **X** = [*x*_1_ *x*_2_]^T^ is the network input vector; $K=[\begin{array}{cc} k_{11} & k_{12}\\ k_{21} & k_{22}\\ \vdots & \vdots \\ k_{m1} & k_{m2} \end{array}]$ is the connection weight matrix from the input layer to the hidden layer; $b={\left[\begin{array}{cccc} b_{1} & b_{2} & \cdots & b_{m} \end{array}\right]}^{T}$ is the hidden layer unit threshold vector; $w_{b}={\left[\begin{array}{cccc} w_{1} & w_{2} & \cdots & w_{m} \end{array}\right]}^{T}$ is the unit connection weight matrix (vector) from the hidden layer to the output layer of the original function network; *c* is the output layer threshold scalar of the original function network. The structure of the original function network is shown in Fig 8.

(2) Constructing the integrand network

The integrand network can be obtained by taking the derivative of a and b in Eq (6). The vector form of the function relationship between the output and input variables of the integrand function network can be written as

$$y=w_{a}^{T}f(KX+b).$$ (7)

**Fig 9:**
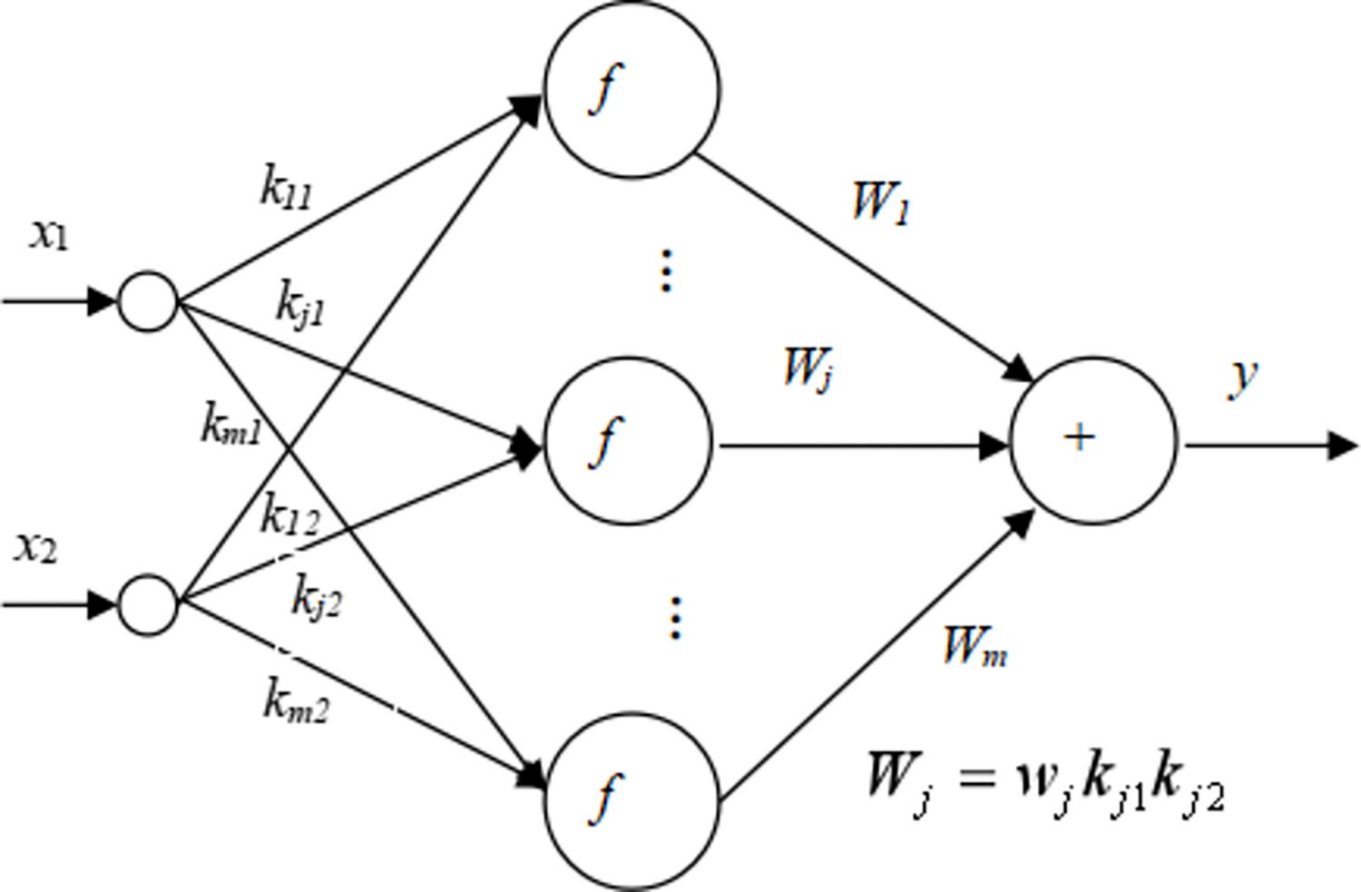
The integrand network structure.

The integrand network is also a three-layer neural network. The vectors **K** and **b** remain unchanged after the derivation because the activation function of the hidden layer is an exponential function. The unit connection weight matrix (vector) from the hidden layer to the output layer is $w_{a}={\left[\begin{array}{cccc} w_{1}k_{11}k_{12} & w_{2}k_{21}k_{22} & \cdots & w_{m}k_{m1}k_{m2} \end{array}\right]}^{T}$. The structure of the integrand network is shown in Fig 9.

(3) Training the integrand network

The linear expansion coefficient *x*_1_ and the thermal conductivity coefficient *x*_2_ are divided by 50 in the interval [*μ*_*i*_−5*σ*_*i*_,*μ*_*i*_+5*σ*_*i*_]. Two nodes cross to form the input sample points of the integrand network. The integrand function is used to obtain the network output value of the corresponding sample point. The Levenberg–Marquardt training algorithm is used to train the integrand network and obtain the network weight and threshold.

(4) Using the primitive function network to solve reliability

The original function network can be obtained by using the dual neural network relationship. The original function is expressed as *Y*(*x*_1_,*x*_2_). The sample of each vertex in the hypercube of the integrand function network is extracted, and the simulation calculation is performed through the original function network. The integral value can be expressed as

$$J=\sum_{m_{1}=1}^{2}\sum_{m_{2}=1}^{2}{\left(-1\right)}^{\sum_{k=1}^{2}m_{k}}Y(x_{1}^{m_{1}},x_{2}^{m_{2}}).$$ (8)

Then, the reliability of the grain material can be solved.

### Result analysis

The structural reliability calculation results of the proposed method and the MCS method when the calculation times of finite element analysis are 20, 50, 100, and 200 are listed in Table 4. The results are compared with the results of 10000 MCS sampling calculations. The table shows that the method in this study can achieve a high calculation accuracy when the finite element analysis times are the same. In addition, only a few analysis times can achieve a high calculation accuracy in this example. The above analysis verifies the feasibility of the proposed method in engineering problems.

**Table 4:** Comparison of results among the proposed and MCS methods.

Calculation times	MCS method	Relative error (%)	Methods in this study	Relative error (%)
20	0.99951	3.7105	0.93121	3.3764
50	0.99426	3.1658	0.93965	2.5006
100	0.99355	3.0921	0.94174	2.2838
200	0.99108	2.8358	0.94893	1.5377
10000	0.96375	-	-	-

## Conclusions

This study analyzes the correlation between linear expansion coefficient and thermal conductivity based on the proposed dual neural network numerical integration method and copula function. The instantaneous reliability of the dangerous point at the dangerous moment is obtained when the grain is solidified and cooled. The instantaneous reliability of the grain is calculated during solidification cooling to measure its reliability in the whole process. This approach can reduce the calculation cost of the grain material’s dynamic reliability. The comparison of the structural reliability results of the proposed method with those of the MCS method shows that the proposed method can achieve a high calculation accuracy.

For practical engineering problems, the analytical expressions of the function functions are often unavailable. In this case, the finite element method is often used to solve the problems mentioned. In this study, the finite element calculation platform ANSYS software is used to calculate the grain’s structural response. Then, the allowable response is combined with the dual neural network integration method and the copula function method to achieve the grain’s structural reliability. This method provides an effective means to solve practical engineering problems.
